# PINK1-Parkin Pathway Activity Is Regulated by Degradation of PINK1 in the Mitochondrial Matrix

**DOI:** 10.1371/journal.pgen.1004279

**Published:** 2014-05-29

**Authors:** Ruth E. Thomas, Laurie A. Andrews, Jonathon L. Burman, Wen-Yang Lin, Leo J. Pallanck

**Affiliations:** 1 Department of Genome Sciences, University of Washington, Seattle, Washington, United States of America; 2 Department of Biology, University of Washington, Seattle, Washington, United States of America; University of California Los Angeles, United States of America

## Abstract

Loss-of-function mutations in *PINK1*, which encodes a mitochondrially targeted serine/threonine kinase, result in an early-onset heritable form of Parkinson's disease. Previous work has shown that PINK1 is constitutively degraded in healthy cells, but selectively accumulates on the surface of depolarized mitochondria, thereby initiating their autophagic degradation. Although PINK1 is known to be a cleavage target of several mitochondrial proteases, whether these proteases account for the constitutive degradation of PINK1 in healthy mitochondria remains unclear. To explore the mechanism by which PINK1 is degraded, we performed a screen for mitochondrial proteases that influence PINK1 abundance in the fruit fly *Drosophila melanogaster*. We found that genetic perturbations targeting the matrix-localized protease Lon caused dramatic accumulation of processed PINK1 species in several mitochondrial compartments, including the matrix. Knockdown of Lon did not decrease mitochondrial membrane potential or trigger activation of the mitochondrial unfolded protein stress response (UPR^mt^), indicating that PINK1 accumulation in Lon-deficient animals is not a secondary consequence of mitochondrial depolarization or the UPR^mt^. Moreover, the influence of Lon on PINK1 abundance was highly specific, as Lon inactivation had little or no effect on the abundance of other mitochondrial proteins. Further studies indicated that the processed forms of PINK1 that accumulate upon Lon inactivation are capable of activating the PINK1-Parkin pathway *in vivo*. Our findings thus suggest that Lon plays an essential role in regulating the PINK1-Parkin pathway by promoting the degradation of PINK1 in the matrix of healthy mitochondria.

## Author Summary

Mitochondria are essential organelles that provide most of the cell's energy and perform many other critical functions. The gradual accumulation of defective mitochondria is thought to play a role in aging and in diseases of the nervous system, including Parkinson's disease. The selective elimination of defective mitochondria is therefore a vital task for the cell, and the protein PINK1 was recently identified as a critical player in this process. PINK1 accumulates on the surface of mitochondria after they are damaged, starting a process that leads ultimately to the elimination of defective mitochondria. Previous work indicated that PINK1 does not accumulate on healthy mitochondria because it is rapidly degraded. However, it was unclear exactly how and where this degradation occurred. Our work shows that Lon protease promotes the degradation of PINK1 in the mitochondrial matrix. This finding provides new insight into the mechanisms of mitochondrial quality control, and reveals a potential strategy for treating the many diseases associated with the accumulation of defective mitochondria.

## Introduction

The accumulation of defective mitochondria is strongly implicated in aging, as well as a variety of common age-related diseases [1], [2], [3]. To counteract this accumulation, cells have evolved a number of mitochondrial quality control pathways. While previous work has revealed molecular mechanisms involved in the prevention and repair of mitochondrial damage [4], [5], [6], the mechanism by which defective mitochondria are selectively detected and degraded was unknown until relatively recently. Over the past several years, studies of the *PTEN-induced putative kinase 1* (*PINK1*) and *parkin* genes, loss-of-function mutations of which give rise to heritable forms of Parkinson's disease [7], [8], have demonstrated that these genes encode components of a mitochondrial quality control system that promotes the selective degradation of defective mitochondria [9], [10], [11]. These studies have led to a model in which PINK1, a mitochondrially localized serine/threonine kinase, is constitutively degraded in cells with healthy mitochondria, but is selectively stabilized on the outer membrane of defective mitochondria. The accumulated PINK1 then recruits the cytosolic E3 ubiquitin ligase Parkin, which ubiquitinates proteins on the mitochondrial surface, leading to isolation of the defective mitochondria and their eventual degradation in the lysosome [9], [10], [11].

Although studies of the PINK1-Parkin pathway have dramatically advanced our understanding of the mechanisms underlying mitochondrial quality control, many questions remain unanswered. One of the most important of these questions concerns the mechanism of PINK1 degradation by healthy mitochondria. Constitutive elimination of PINK1 prevents healthy mitochondria from being inappropriately destroyed, but permits a rapid response when PINK1 degradation ceases due to mitochondrial dysfunction. Previous work has identified three mitochondrially localized proteases that appear to participate in PINK1 proteolytic processing: mitochondrial processing peptidase (MPP) [12]; AFG3-like AAA ATPase 2 (AFG3L2) [12]; and Rhomboid-7/Presenilin-associated rhomboid-like protein, mitochondrial (Rho-7/PARL) [13], [14]. MPP removes the N-terminal mitochondrial targeting sequence of PINK1 and many other mitochondrial proteins [15]. The processing event mediated by AFG3L2 is unknown, although previous work suggests that AFG3L2 may facilitate the Rho-7/PARL cleavage event [12]. Rho-7/PARL cleaves PINK1 within its transmembrane domain [14], [16], creating a form of PINK1 that is released to the cytosol and degraded by the proteasome [17], [18], [19]. However, not all PINK1 degradation is dependent on Rho-7/PARL [12], [13], [14], suggesting that there are other mechanisms of PINK1 degradation.

To explore the mechanisms by which PINK1 is degraded, we used the fruit fly *Drosophila melanogaster* to conduct a screen for mitochondrial proteases that affect PINK1 processing and stability *in vivo*. Our work indicates that PINK1 is directed to the mitochondrial matrix, where it is degraded by the matrix-localized Lon protease. We also found that inactivation of Lon leads to the accumulation of cleaved forms of PINK1 that are active *in vivo*. Thus, Lon appears to represent a critical component of the mitochondrial proteolytic machinery that opposes PINK1 accumulation on the surface of healthy mitochondria.

## Results

### Lon protease influences PINK1 abundance

To identify proteases that influence PINK1 stability, we used the pan-neuronal driver *elav-GAL4* to express RNA interference (RNAi) constructs targeting known mitochondrial proteases in a fly strain that also bears a transgene with a *myc*-tagged form of *PINK1* (Table S1). To avoid overexpression artifacts associated with the UAS/GAL4 system, we used a transgenic line that expresses PINK1-Myc under the control of the endogenous *PINK1* promoter [20]. Flies bearing this transgene have only a small increase in PINK1 expression and exhibit no detectable abnormalities (Fig. S1 and data not shown). We then performed anti-Myc immunoblotting on head protein extracts from flies co-expressing the PINK1 transgene and the GAL4-driven RNAi constructs to assess the effects of protease knockdown on PINK1 processing and abundance. The anti-Myc antiserum detected four bands in protein extracts from flies expressing a control RNAi targeting an exogenous gene (*mCherry*, hereafter Control-R) (Fig. S2A). We propose that the highest molecular weight (MW) PINK1 band corresponds to unprocessed, full-length PINK1 (FL-PINK1). The second highest MW PINK1 band differs from the highest MW band by a mass consistent with that of the predicted PINK1 mitochondrial targeting sequence, so we propose that this band is the MPP-processed form of PINK1 (MPP-PINK1). The third highest MW PINK1 band corresponds in size to the Rho-7/PARL–processed form of PINK1 (Rho-PINK1), as identified previously [13]. The origin of the lowest MW PINK1 band is uncertain, but appears to be dependent on the AFG3L2 protease, as this band was nearly absent in AFG3L2-deficient animals (Fig. S2A, B). This finding led us to name the lowest PINK1 band AFG-PINK1. Among the 13 mitochondrial proteases tested in our study, only RNAi constructs targeting *AFG3L2* (*CG6512*) and *Lon protease* (*CG8798*) resulted in accumulation of PINK1 (Table S1). For the remainder of our studies we focused on Lon as Lon involvement in PINK1 processing had not been previously characterized.

**Figure 1:**
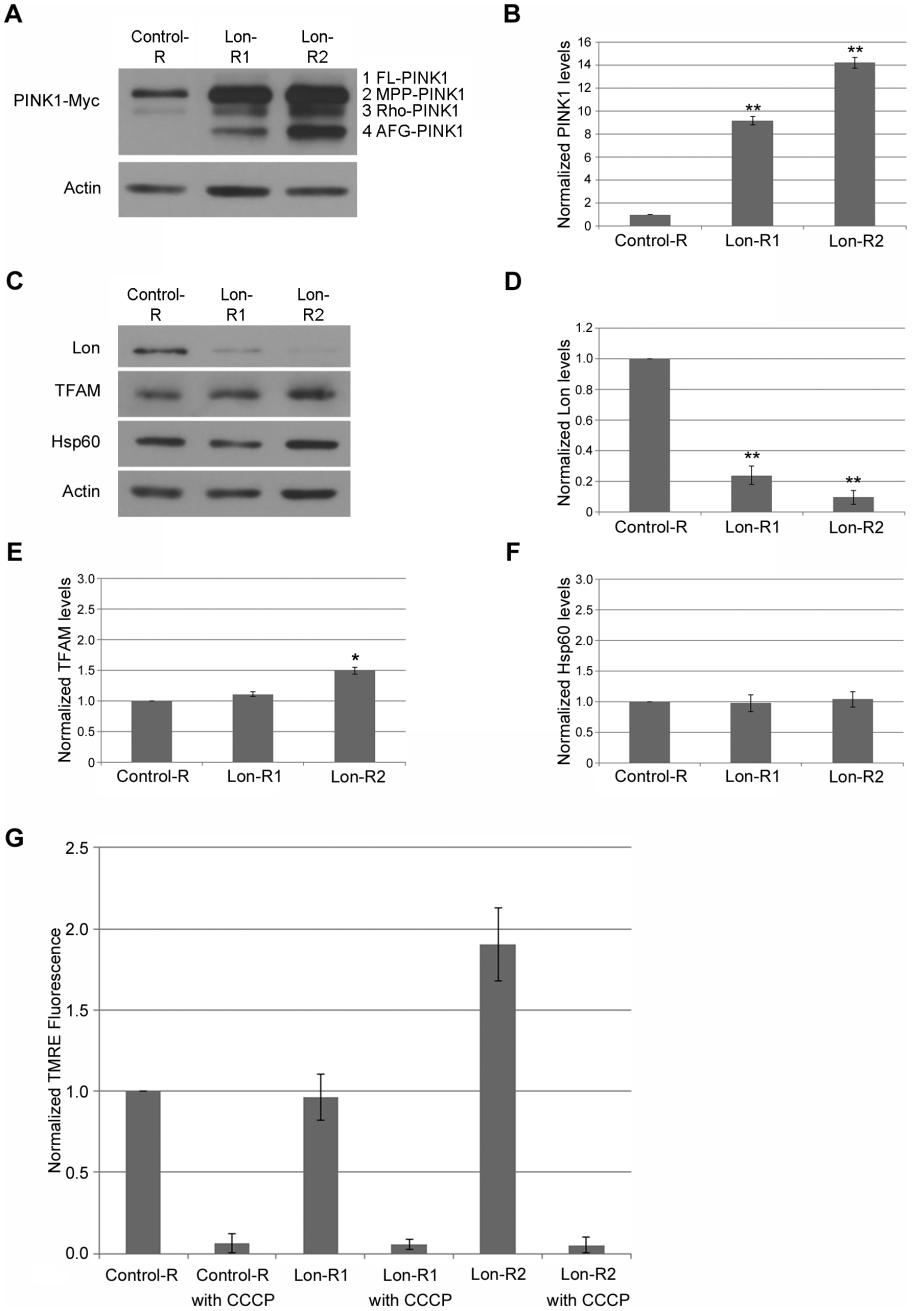
PINK1 accumulates upon knockdown of Lon. (A) Western blot analysis of whole head homogenates from transgenic flies expressing a Myc-tagged PINK1 genomic construct and a UAS-RNAi construct driven by *elav-GAL4*. The RNAi constructs used were as follows: the non-specific control *mCherry*-RNAi (Control-R), *Lon*-RNAi1 (Lon-R1), and *Lon*-RNAi2 (Lon-R2). An anti-Myc antibody was used to detect PINK1 and an anti-Actin antibody was used as a protein loading control. (B) Densitometry of the PINK1-Myc bands from the indicated genotypes was performed using Fiji software. The PINK1-Myc band intensities were then normalized to their respective Actin loading controls, and these ratios were in turn normalized to the Control-R PINK1/Actin ratio. (C) Western blot analysis of Lon protease (Lon), Mitochondrial transcription factor A (TFAM), Heat shock protein 60 (Hsp60) and Actin in whole head homogenate from control and Lon deficient animals. (D) Quantification of the Lon band intensities from the indicated genotypes performed as described in B. (E) Quantification of the TFAM bands from the indicated genotypes performed as described in B. (F) Quantification of the Hsp60 bands from the indicated genotypes performed as described in B. (G) Flow cytometry analysis of dissociated cells from the brains of flies expressing the indicated RNAi constructs. The graph shows the normalized fluorescence intensity of TMRE, an indicator of mitochondrial membrane potential, relative to age-matched control animals. Fluorescence intensity was normalized to control samples prepared and analyzed on the same day as experimental samples. The mitochondrial membrane potential uncoupling agent CCCP was added to samples of the indicated genotypes to illustrate the effect of mitochondrial depolarization on TMRE signal intensity. All experiments described in this figure were repeated at least three times per genotype. Error bars represent the standard error of the mean (s.e.m.). *p<0.05, **p<0.005 by Student *t* test.

RNAi-mediated knockdown of Lon led to a dramatic accumulation of all three processed PINK1 isoforms (Fig. 1A, B). Importantly, the effects of Lon knockdown were reproduced with two independent *Lon* RNAi constructs, and the magnitude of PINK1 accumulation correlated with the extent of Lon knockdown (Fig. 1B–D), indicating that PINK1 accumulation is a consequence of Lon inactivation, rather than an RNAi off-target effect. Thus, our finding that MPP-PINK1, Rho-PINK1, and AFG-PINK1 accumulate upon Lon inactivation raises the possibility that these PINK1 isoforms are substrates of Lon-mediated matrix degradation. However, before testing this model further, we explored other possible explanations of our findings.

**Figure 2:**
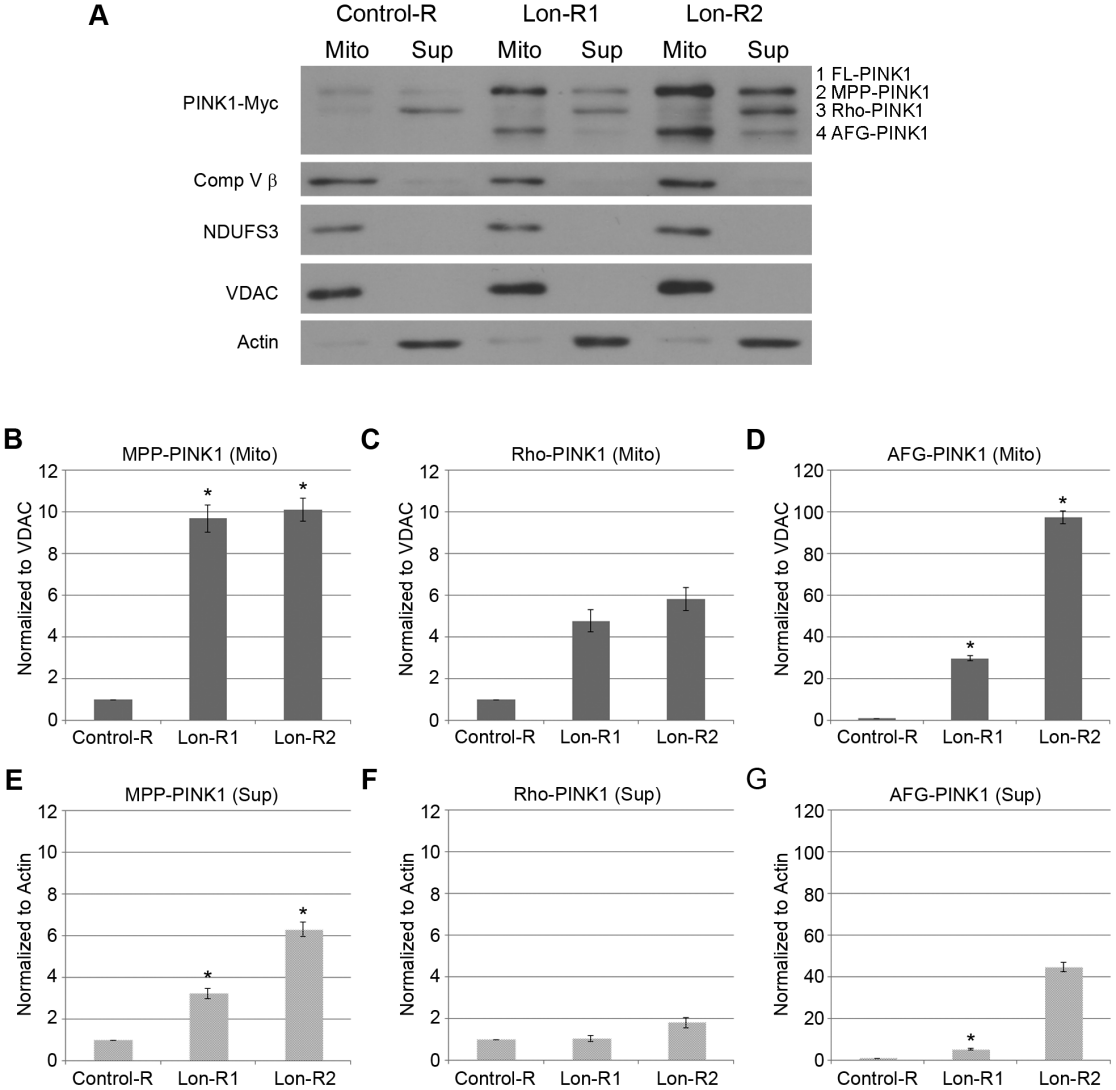
PINK1 accumulates in the mitochondrial and postmitochondrial supernatant fractions upon knockdown of Lon. (A) Mitochondrial (Mito) and postmitochondrial supernatant (Sup) protein fractions isolated from the heads of flies of the indicated genotypes were analyzed by western blot with antibodies to Myc, Complex V β (Comp V β), NADH dehydrogenase (ubiquinone) Fe-S protein 3 (NDUFS3), voltage-dependent anion channel (VDAC) and Actin. *elav-GAL4* was used to drive expression of the RNAi constructs used in these experiments. (B) Densitometry of the MPP-PINK1 bands in the mitochondrial fractions from the indicated genotypes was performed using Fiji software. PINK1 band intensities were normalized to VDAC intensity, and this ratio was in turn normalized to the MPP-PINK1/VDAC ratio in Control-R animals. (C) Quantification of the Rho-PINK1 band intensities in mitochondrial fractions from the indicated genotypes was performed as described in B. (D) Quantification of the AFG-PINK1 band intensities in mitochondrial fractions from the indicated genotypes was performed as described in B. (E) Quantification of the MPP-PINK1 band intensities in the supernatant fractions from the indicated genotypes was performed as described in B except that Actin rather than VDAC was used as the loading control. (F) Quantification of the Rho-PINK1 band intensities in supernatant fractions from the indicated genotypes was performed as described in E. (G) Quantification of the AFG-PINK1 band intensities in supernatant fractions from the indicated genotypes was performed as described in E. Results shown are representative of at least three independent subcellular fractionation experiments per genotype. Error bars represent s.e.m. *p<0.05 by Student *t* test.

Lon has been shown to degrade a number of mitochondrial proteins [21], [22], and is also implicated in mitochondrial DNA (mtDNA) stability [23], [24], [25] and in the mitochondrial unfolded protein stress response [26]. Recently published work also suggests that mitochondrial unfolded protein stress triggers activation of the PINK1-Parkin pathway [27]. Thus, PINK1 accumulation in response to Lon knockdown could be a downstream consequence of a generalized matrix protein degradation defect, mtDNA instability, or the mitochondrial unfolded protein response (UPR^mt^). However, knockdown of Lon did not influence the abundance of the inner membrane–associated matrix proteins Complex V β (Comp V β) or NADH dehydrogenase (ubiquinone) Fe-S protein 3 (NDUFS3) (Fig. 2A), and only mildly affected the abundance of a known Lon substrate, Mitochondrial transcription factor A (TFAM). Moreover, the effect of Lon knockdown on TFAM abundance was only seen in flies expressing the stronger *Lon* RNAi construct, Lon-R2 (Fig. 1E). Knockdown of Lon also had no effect on mtDNA abundance (Fig. S3), or on the abundance of Heat shock protein 60 (Hsp60), a marker of the UPR^mt^ (Fig. 1C, F). Thus, PINK1 accumulation appears to be a relatively specific consequence of Lon inactivation rather than a downstream consequence of a general matrix protein degradation defect, mtDNA instability, or UPR^mt^ activation.

Because PINK1 accumulates in cell culture upon mitochondrial depolarization [10], [11], another potential explanation of our findings is that Lon inactivation triggers mitochondrial depolarization. To explore this possibility, we used a recently described procedure to measure mitochondrial membrane potential in the adult *Drosophila* nervous system [28]. Briefly, we dissociated the brains from flies expressing either a control RNAi or RNAi targeting *Lon* to create neural cell suspensions. We then stained the cell suspensions with the mitochondrial membrane potential–dependent dye tetramethylrhodamine, ethyl ester (TMRE), and used flow cytometry to compare the distribution of mitochondrial membrane potential in these cell populations. Neither of the RNAi constructs targeting *Lon* caused a significant reduction of mitochondrial membrane potential (Fig. 1G). Rather, there was a trend for the stronger *Lon* RNAi construct, Lon-R2, to cause hyperpolarization. These findings indicate that the increased PINK1 abundance upon Lon inactivation is not due to decreased mitochondrial membrane potential. We therefore performed additional studies to explore the role of Lon in PINK1 processing.

### Lon promotes PINK1 degradation in the mitochondrial matrix

Because Lon is a matrix-localized protease, the simplest interpretation of our findings is that Lon promotes the degradation of PINK1 in the mitochondrial matrix. This model predicts that Lon inactivation should result in the accumulation of PINK1 in the matrix. To test this model, we first examined the subcellular localization of the accumulated PINK1 isoforms following knockdown of Lon, using differential sedimentation to generate mitochondrial and postmitochondrial supernatant fractions. Previous work has shown that Rho-PINK1 can be released into the cytosol, where it is degraded by the proteasome [17], [18], [19]. Consistent with this work, we found that most of the PINK1 protein in control animals consisted of Rho-PINK1 in the supernatant fraction (Fig. 2A). We also detected lesser amounts of Rho-PINK1 in the mitochondrial fraction, and faint bands of MPP-PINK1 in the mitochondrial and the postmitochondrial supernatant fractions of control animals, suggesting that MPP-PINK1 can also be released from mitochondria (Fig. 2A). RNAi-mediated inactivation of *Lon* resulted in the accumulation of all processed forms of PINK1, including AFG-PINK1, in both the mitochondrial and postmitochondrial supernatant fractions, particularly in the case of the stronger Lon-R2 construct (Fig. 2). However, by far the largest increases in PINK1 accumulation upon Lon inactivation were seen in the mitochondrial fraction (compare 2B–D with 2E–G), consistent with the model that Lon promotes PINK1 degradation in the mitochondrial matrix. The finding that Lon inactivation also results in the accumulation of PINK1 in the postmitochondrial supernatant fraction raises the possibility that Lon is also required for the efficient import of PINK1 into the matrix.

**Figure 3:**
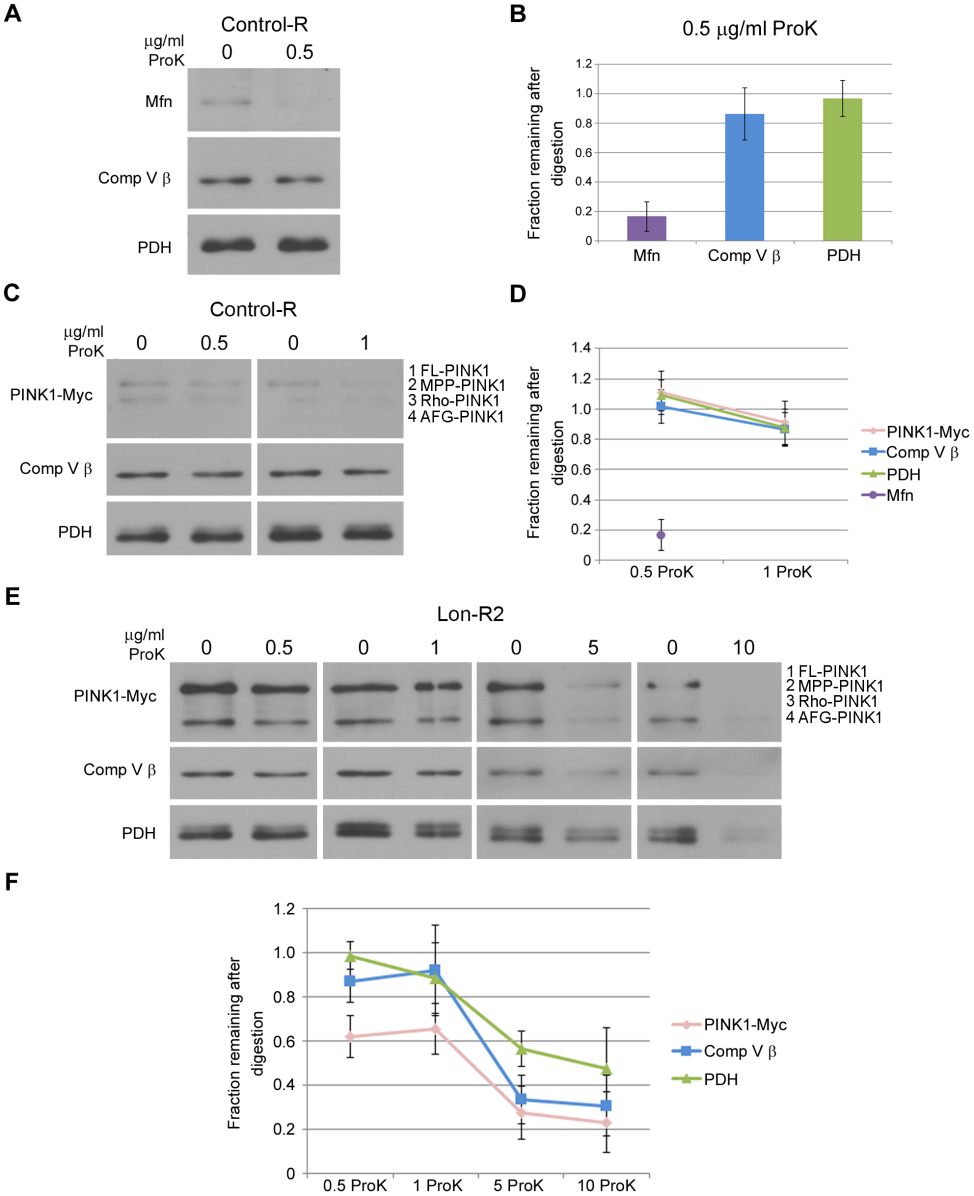
PINK1 accumulates on the outer membrane and in the matrix upon knockdown of Lon. (A) Mitochondrial fractions isolated from the heads of flies expressing Control-R were divided in half. One of the two samples was treated with 0.5 µg/ml Proteinase K and both samples were incubated at 4°C for 20 minutes. Samples were then subjected to western blot analysis using antibodies to the outer membrane protein Mitofusin (Mfn), the inner membrane–associated matrix protein Complex V β (Comp V β), and the matrix luminal protein pyruvate dehydrogenase (PDH). *elav-GAL4* was used to drive expression of all the RNAi constructs used in this and the following experiments. (B) Densitometry of Mfn, Comp V β, and PDH bands from experiments represented by panel A were performed using Fiji software, and the ratios of the band intensities in the sample containing ProK to the sample lacking ProK are shown. (C) Mitochondrial fractions isolated from the heads of flies expressing Control-R and PINK1-Myc were divided in half. Each sample was treated with the indicated concentrations of ProK. Following incubation, samples were subjected to western blot analysis using antibodies to the indicated proteins. (D) Densitometry of the PINK1-Myc, Comp V β, and PDH bands from experiments represented by panel C were performed as described in B. The Mfn quantification data from panel B is also included for comparison. (E) Mitochondrial fractions isolated from the heads of Lon-R2 and PINK1-Myc expressing flies were divided in half. Each sample was treated with the indicated concentrations of ProK. Following incubation, samples were subjected to western blot analysis using antibodies to the indicated proteins. (F) Densitometry of the PINK1-Myc, Comp V β, and PDH bands from experiments represented by panel E were performed as described in B. All experiments described were repeated at least three times. Error bars represent s.e.m.

To test whether the PINK1 that accumulates in mitochondrial fractions from Lon-deficient animals resides in the matrix, we performed protease protection experiments on isolated mitochondria. Specifically, we compared the Proteinase K (ProK) digestion sensitivities of the various PINK1 isoforms to the sensitivities of Mitofusin (Mfn), Complex V β (Comp V β), and pyruvate dehydrogenase (PDH), which reside on the outer mitochondrial membrane, on the matrix side of the inner membrane, and in the matrix lumen, respectively. Treating a mitochondrial fraction from control flies with a low concentration of ProK (0.5 µg/ml) resulted in substantial degradation of Mfn but did not significantly influence the abundance of Comp V β, PDH, or PINK1 (Fig. 3A–D). However, disrupting mitochondrial membranes with Triton X-100 followed by ProK treatment at a low concentration (0.5 µg/ml) resulted in nearly complete degradation of Comp V β, PDH, and PINK1 in both control and Lon-deficient animals (Fig. S4), indicating that the resistance of these proteins to ProK degradation in intact mitochondria (Fig. 3A–D) is a consequence of their internal localization rather than of inherent ProK insensitivity. Upon treatment of mitochondria from Lon-deficient animals with a low concentration (0.5 µg/ml) of ProK, the MPP-PINK1 and AFG3-PINK1 isoforms were 40% depleted (Fig. 3E, F). The remaining 60% of MPP-PINK1 and AFG-PINK1 in mitochondrial fractions from Lon-deficient animals was substantially depleted only at ProK concentrations that also resulted in depletion of Comp V β and PDH (5 and 10 µg/ml; Fig. 3F). These findings indicate that PINK1 accumulates in both the outer membrane and the matrix upon Lon inactivation, consistent with the model that Lon protease promotes the import and degradation of PINK1 in the mitochondrial matrix.

**Figure 4:**
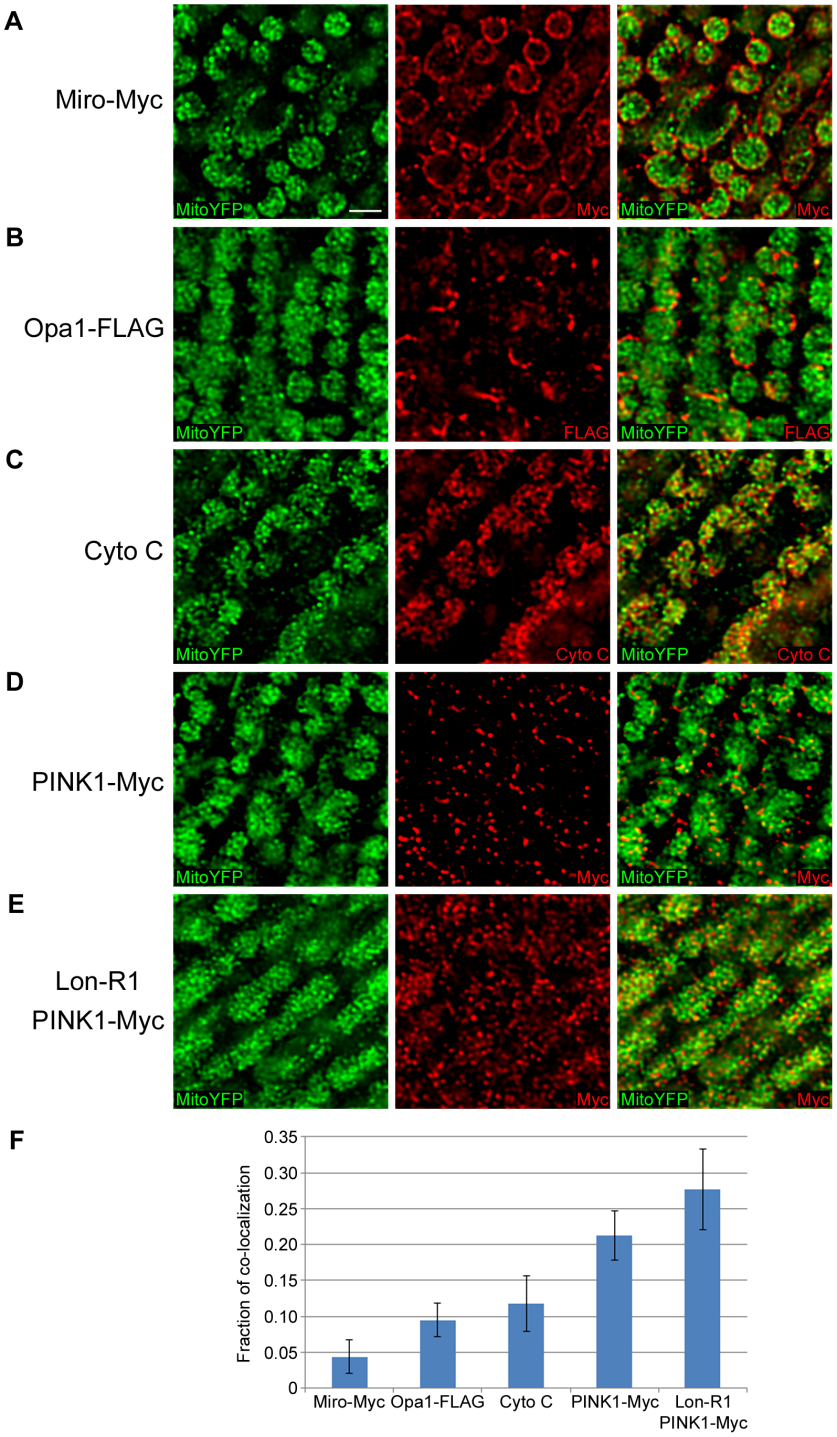
PINK1 accumulates in the mitochondrial matrix upon knockdown of Lon. Immunofluorescent staining of thoracic muscles expressing matrix-targeted YFP (Mito-YFP) under control of the *sqh* genomic promoter, together with either (A) Myc-tagged Mitochondrial Rho (Miro-Myc), (B) FLAG-tagged Optic atrophy 1 (Opa1-FLAG), (C) endogenous Cytochrome C (Cyto C), (D) Myc-tagged PINK1 (PINK1-Myc), or (E) *Lon*-RNAi1 (Lon-R1) and PINK1-Myc. The muscle-specific *Mhc-GAL4* driver was used to drive expression of Miro-Myc in panel A and Opa1-FLAG in panel B, and the muscle-specific *Dmef2-GAL4* driver was used to drive expression of Lon-R1 in panel E. (F) We determined the percent volume shared between green objects (representing the matrix marker Mito-YFP) and red objects (representing the mitochondrial proteins analyzed in A–E). Red objects that shared at least 60% of their volume with Mito-YFP were considered to co-localize. Bar represents 2 µm.

To confirm our finding that at least some of the PINK1 that accumulates in Lon-deficient animals resides in the mitochondrial matrix, we used confocal microscopy to examine the localization of PINK1 following Lon knockdown. We performed this experiment using thoracic muscle because the large mitochondria in this tissue are relatively easy to image. We compared the localization patterns of a mitochondrial matrix–targeted YFP (Mito-YFP) to that of a Myc-tagged form of Mitochondrial Rho (Miro-Myc), a FLAG-tagged form of Optic atrophy 1 (Opa1-FLAG), endogenous cytochrome c (Cyto C), and PINK1-Myc (Fig. 4). Previous work has established that Miro localizes to the mitochondrial outer membrane [29], Opa1 to the inner membrane [30], and Cyto C to the intracristal space [31]. The Mito-YFP showed the characteristic, highly invaginated structure of the matrix. The Miro-Myc signal surrounded the Mito-YFP signal, as would be expected for an outer mitochondrial membrane protein (Fig. 4A). Opa1-FLAG also surrounded the Mito-YFP and interleaved with it, as would be predicted for a protein localized to the inner membrane, particularly the necks of the cristae [30] (Fig. 4B). Cyto C interleaved with the Mito-YFP, consistent with its localization within cristae [31] (Fig. 4C). When we examined the localization of PINK1-Myc in control flies, we detected only a small amount of PINK1 signal, consistent with previous work demonstrating that PINK1 is rapidly degraded in healthy mitochondria (Fig. 4D) [10], [11]. However, animals expressing the *Lon* RNAi construct exhibited a substantial accumulation of PINK1 that co-localized in part with matrix-targeted Mito-YFP (Fig. 4E). The degree of co-localization between Mito-YFP and the various mitochondrial proteins analyzed in Figure 4 was measured by determining the percentage of volume that the red objects (individual areas of signal from the various mitochondrial proteins) shared with Mito-YFP. Those red objects that shared 60% or more of their volume with Mito-YFP were considered to be co-localized. This analysis revealed that PINK1 in Lon-deficient animals had the highest degree of co-localization with Mito-YFP, followed by PINK1 in control animals, and then Cyto C, Opa1-FLAG, and Miro-Myc in descending order (Fig. 4F). This finding confirms our biochemical observations and offers further support for the model that Lon protease promotes the degradation of PINK1 in the mitochondrial matrix.

### Lon regulates PINK1-Parkin pathway activity *in vivo*

Previous work has shown that the accumulation of PINK1 on the mitochondrial outer membrane triggers the recruitment of Parkin and the eventual degradation of mitochondria [10], [11]. Thus, our finding that some of the excess MPP-processed and AFG3L2-processed PINK1 in Lon-deficient animals accumulates on the mitochondrial outer membrane raises the possibility that Lon inactivation might trigger PINK1-Parkin pathway activity. In potential support of this hypothesis, there is a trend towards increased mitochondrial membrane potential in Lon-deficient animals that mirrors the increased mitochondrial membrane potential seen in flies overexpressing PINK1 [28]. We performed several experiments to test this hypothesis directly.

**Figure 5:**
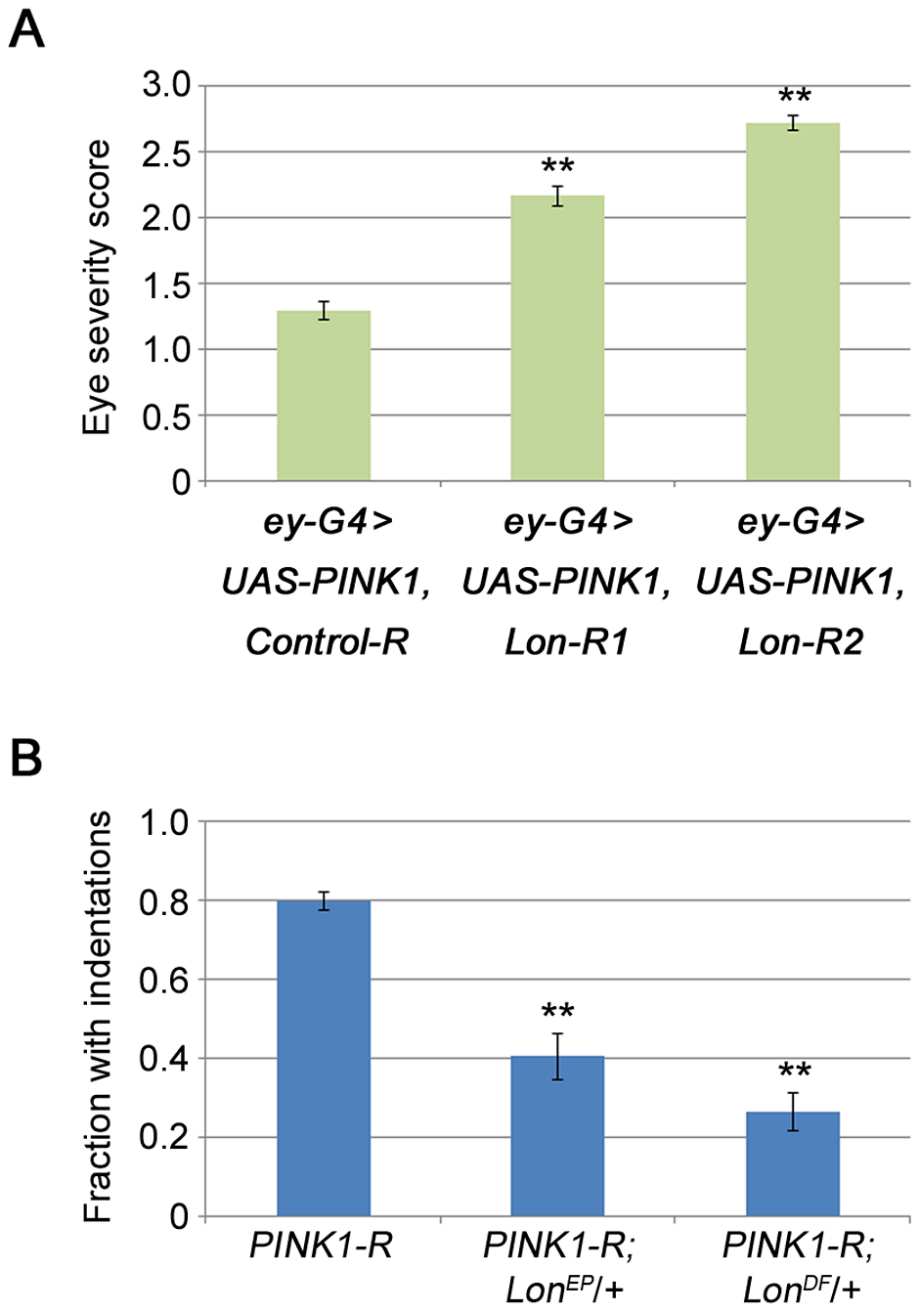
Lon deficiency stimulates PINK1 activity *in vivo*. (A) The eye severity score of flies co-overexpressing PINK1 with Control-R (*n* = 74), Lon-R1 (*n* = 163), or Lon-R2 (*n* = 90) using the *ey-GAL4* driver. (B) Comparison of the frequency of thoracic indentations in flies expressing the *PINK1*-RNAi construct (PINK1-R) in an otherwise wild-type background to the frequency in animals that also bear heterozygous mutations in *Lon*: *PINK1*-RNAi +/+ (*n* = 306), *PINK1*-RNAi *Lon^EP^*/+ (*n* = 74), and *PINK*1-RNAi *Lon^Df^*/+ (*n* = 83). *Lon^EP^* refers to the *Lon^G3998^* P-element allele of *Lon*, and *Lon^Df^* refers to the *Df(3L)Exel9011* deletion that removes the *Lon* gene along with several other genes. The *Dmef2-GAL4* driver was used to drive expression of the PINK1-R construct in experiments described in panel B. Error bars represent s.e.m. *p<0.05, **p<0.005 by Student *t* test.

To determine whether inactivation of Lon influences PINK1-Parkin pathway activity *in vivo*, we first explored the influence of Lon deficiency on a PINK1 overexpression phenotype. We have previously shown that overexpressing PINK1 in a variety of *Drosophila* tissues is toxic, and that this toxicity can be suppressed by homozygous loss-of-function mutations in *parkin* and dramatically enhanced by co-overexpression of Parkin [32]. These and other findings indicate that the toxicity associated with PINK1 overexpression results from an amplification of PINK1-Parkin pathway activity. A convenient way to quantify this toxicity involves monitoring the extent to which the structure of the compound eye becomes disorganized when PINK1 is overexpressed in the eye using the *ey-GAL4* driver. We used this approach to test the influence of Lon on PINK1-Parkin pathway activity. Both RNAi constructs targeting *Lon* significantly enhanced (worsened) the PINK1 overexpression phenotype (Fig. 5A), as would be predicted if Lon normally acts to restrict PINK1 activity. Moreover, the enhancement produced by these RNAi constructs correlated with their efficiency in reducing Lon expression, and with their effectiveness in triggering PINK1 accumulation (Fig. 1A, B). Neither of the *Lon* RNAi constructs detectably influenced eye structure when driven with the *ey-GAL4* driver in an otherwise WT background (Fig. S5), suggesting that the enhancement of the PINK1 overexpression phenotype conferred by these RNAi constructs is not simply an additive effect of combining two unrelated perturbations that affect the eye.

Next we examined the effects of a P-element insertion and a deletion targeting *Lon* on a thoracic indentation phenotype produced by PINK1 deficiency in the flight muscle. Previous work has shown that thoracic indentations serve as a reliable surrogate marker of muscle pathology in *PINK1* and *parkin* mutants [20], [32], [33], [34]. We used an RNAi line targeting *PINK1* that yields a hypomorphic phenotype, because decreases in Lon activity would not be expected to improve a *PINK1* null phenotype. We also avoided the use of RNAi constructs targeting *Lon* because the introduction of any additional UAS element suppressed the thoracic indentation phenotype caused by *PINK1* RNAi, likely through a GAL4 dilution effect (data not shown). We found that a heterozygous P-element insertion mutation in *Lon* suppressed the *PINK1*-RNAi thoracic indentation phenotype (Fig. 5B) but did not influence the thoracic indentation frequency of *PINK1* null mutants, confirming that Lon deficiency suppresses the phenotype only when some PINK1 is present (Fig. S6). Additionally, a heterozygous deletion that completely removes *Lon* caused even stronger suppression (Fig. 5B). Together, our findings indicate that the PINK1 isoforms that accumulate upon knockdown of Lon are capable of triggering PINK1-Parkin pathway activity *in vivo*, and demonstrate that Lon plays an important role in regulating the PINK1-Parkin pathway.

## Discussion

**Figure 6:**
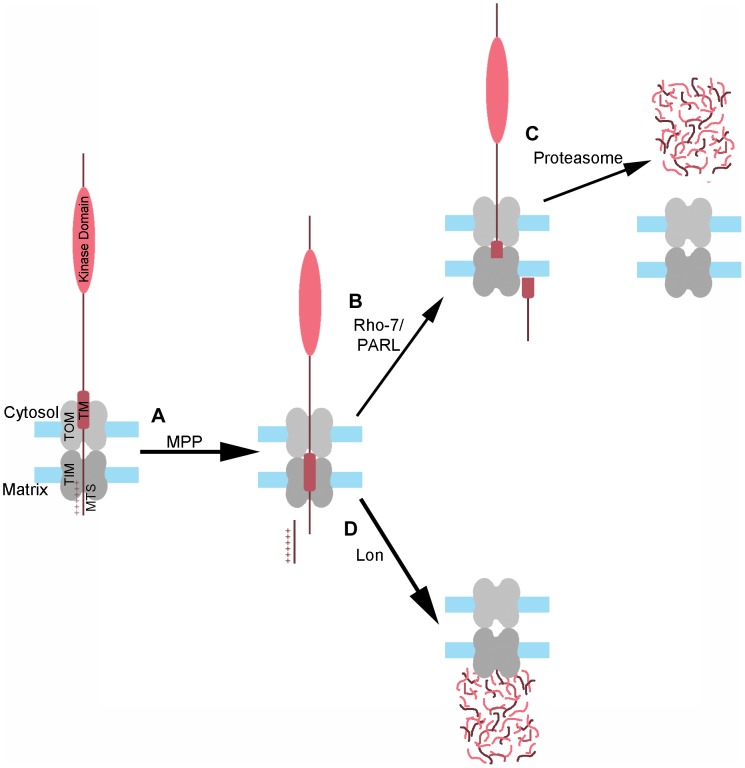
Model of PINK1 processing and degradation. (A) As PINK1 begins to import into mitochondria, the positively charged mitochondrial targeting sequence (MTS) of PINK1 reaches the matrix through the TOM-TIM complex, where MPP recognizes and cleaves this sequence. The MPP-PINK1 cleavage product can then be released into the cytosol, or be further processed within the mitochondria in one of two ways: (B) Rho-7/PARL–mediated cleavage of MPP-PINK1 within the PINK1 transmembrane (TM) domain results in the release of Rho-PINK1 into the cytosol, where it is then degraded by the proteasome (C). Alternatively, (D) MPP-PINK1 is imported into the matrix and degraded by Lon, either directly or following AFG3L2-dependent cleavage of MPP-PINK1 to AFG-PINK1.

Previous work has shown that PINK1 protein is normally maintained at low levels in healthy cells [10], [11]. While the proteasome appears to be responsible for the degradation of a cytosolic Rho-7/PARL–processed form of PINK1 [17], [18], and thus at least partially accounts for the low abundance of PINK1 in healthy cells (Fig. 6A–C), whether PINK1 degradation also occurred in mitochondria remained unclear from previous work. Our study identifies Lon as a protease involved in PINK1 processing and degradation in the mitochondrial matrix (Fig. 6D). Lon may also serve to assist the import of PINK1 into the matrix, given that MPP-PINK1 and AFG-PINK1 accumulate on the mitochondrial surface and in the cytosol upon Lon inactivation. Lon could facilitate PINK1 import into the matrix through its known unfoldase activity, ultimately delivering PINK1 to the Lon protease domain for degradation [35]. While a recent study in vertebrate cell culture also identified the mitochondrial matrix protein AFG3L2 as a PINK1 processing protease, the exact role of AFG3L2 in PINK1 processing was not established [12]. Our work suggests that AFG3L2 is responsible for producing a cleaved form of PINK1, AFG-PINK1. Moreover, our finding that AFG-PINK1 accumulates to a greater extent than other PINK1 isoforms upon Lon inactivation (Fig. 2) further suggests that AFG-PINK1 is the preferred substrate for Lon. Together, our findings support a model in which AFG3L2 and Lon play important roles in mitochondrial quality control, by promoting degradation of PINK1 within the mitochondrial matrix to prevent healthy mitochondria from being targeted for mitophagy.

Our finding that Lon plays a role in the degradation of PINK1 contrasts with two recent papers that examined the effect of Lon knockdown on PINK1 levels in cultured cells [12], [27]. Neither Greene et al. [12] nor Jin et al. [27] observed dramatic accumulation of PINK1 when *Lon* was targeted by shRNA. There are several possible explanations for this discrepancy. One potential explanation is that mammalian Lon may be highly efficient at degrading PINK1, such that even a small amount of Lon activity may be sufficient to fully degrade PINK1. Another potential explanation is that the cultured cells were able to compensate for reduced Lon activity through increased cytoplasmic release of Rho-7/PARL-processed PINK1 for proteasomal degradation. It should also be noted that while Greene et al. and Jin et al. did not observe dramatic accumulation of PINK1 upon Lon knockdown, both these groups and others have reported PINK1 accumulation upon MG132 treatment [12], [14], [18], [36]; while MG132 is best known as a proteasome inhibitor, it is also a potent inhibitor of Lon [37]. Moreover, Jin et al. observed PINK1 accumulation upon expression of an unfolded protein targeted to the matrix (a deletion mutant of ornithine carbamoyltransferase, ΔOTC), and PINK1 accumulation was further enhanced by simultaneously knocking down Lon. From these findings Jin et al. posit that when the UPR^mt^ is insufficient to reduce the unfolded protein stress, PINK1 import is inhibited through an unknown mechanism, thus triggering mitophagy. However, an alternative interpretation of their findings is that ΔOTC acts as a competitive inhibitor of PINK1 degradation by Lon, which is known to degrade unfolded proteins [35]. This model would account for both the accumulation of PINK1 seen upon expression of ΔOTC, and the increased accumulation of PINK1 and ΔOTC that is seen when Lon is knocked down. Further studies will be required to distinguish these models.

Our findings also differ from those of previous biochemical studies concluding that PINK1 does not localize to the mitochondrial matrix [38], [39]. However, the conflicting biochemical studies used cells with intact Lon protease. If PINK1 degradation by Lon is rapid and efficient, one would not expect to detect significant amounts of PINK1 in the mitochondrial matrix except when Lon function is impaired. Indeed, our biochemical experiments are in general agreement with previously published work, as we also detected little PINK1 in mitochondrial fractions from WT control animals.

Although previous work indicated that the accumulation of FL-PINK1 on the outer surface of depolarized mitochondria triggers the activation of the PINK1-Parkin pathway [10], [11], it was unclear whether other processed forms of PINK1 could also trigger pathway activation. Our findings demonstrating that Lon inactivation results in the accumulation of MPP-PINK1 and AFG-PINK1 on the outer mitochondrial membrane, and causes increased PINK1-Parkin pathway activity *in vivo*, suggest that one or both of these forms of PINK1 are also capable of activating the PINK1-Parkin pathway. Recent work also suggests that the PARL-processed form of PINK1 can promote pathway activity, as it can associate with mitochondria *in vitro* and promote the recruitment of Parkin [40]. However, because FL-PINK1 appears to be the only form of PINK1 that accumulates upon mitochondrial depolarization [10], processed forms of PINK1 may not normally participate in pathway activity. They may, however, have other important biological roles. In particular, recent studies implicate PINK1 in phosphorylation [41] and selective turnover [42], [43] of matrix-localized proteins; our finding that processed forms of PINK1 localize at least transiently to the matrix raises the possibility that PINK1 directly mediates these processes. Future work will be needed to fully delineate these possibilities, as well as to explore the possible therapeutic benefits of Lon inhibition in treating the many diseases associated with accumulation of defective mitochondria, including Parkinson's disease.

## Materials and Methods

### Fly stocks

*Drosophila* stocks were maintained on standard cornmeal-molasses food at 25°C. The *PINK1-myc* transgenic line, *UAS-PINK1* transgenic line, *UAS-Miro-myc* transgenic line, *Mhc-GAL4* driver line and *Dmef2-GAL4* driver line have been previously described [20], [34], [44], [45], [46]. The following lines were obtained from the Bloomington Drosophila Stock Center: *elav-GAL4*, *ey-GAL4*, *sqh-mito-EYFP*, *Lon^G3998^*, *Df(3L)Exel9011*, *P{VALIUM20-mCherry}attP2* (Control-RNAi), and *P{TRiP.HMS01060}attP2* (*Lon*-RNAi2). The *P{GD11336}-v21860* (*PINK1*-RNAi), *P{KK101663}-v109629* (*AFG3L2*-RNAi1), and *P{GD14030}-v36036* (*Lon*-RNAi1) RNAi lines were obtained from the Vienna Drosophila Resource Center. All other RNAi lines tested were obtained from the stock centers indicated in Table S1.

### qPCR analysis

For measurement of *PINK1* mRNA, total RNA was extracted from *elav-GAL4*; *Control-R*/+ and *elav-GAL4*; *Control-R*/*PINK1-myc* using TRIzol (#15596-026, Life Technologies). Reverse transcription was done using the iScript cDNA Synthesis kit (#170-8890, Bio-Rad) and diluted 1∶50 and 1∶300 before use in qPCR reactions. Primer sequences for *PINK1* and *Rap2l* were obtained from the FlyPrimerBank [47]. Primer pairs PA60267 and PP23832 were used for *PINK1*, and primer pair PP8673 for *Rap2l*. The log_2_ method was used to calculate fold change. *Rap2l* was used as the internal control, as the expression of this gene has been reported as the most invariant across different genotypes and ages [48]. qPCR was performed using Brilliant III Ultra-Fast SYBR Green QPCR Master Mix (#600882, Agilent Technologies) and a Bio-Rad Opticon 2 machine. Mitochondrial and nuclear DNA abundance were measured by using the DNA extraction method and primers described in a published report [49]. qPCR of mitochondrial and nuclear DNA was performed as described above.

### Western blot analysis

Proteins were separated by SDS-PAGE on 10% Tris-acrylamide gels and electrophoretically transferred onto PVDF membranes. Immunodetections with commercial antibodies were performed at the following concentrations: 1∶1000 mouse anti-Myc 9E10 (#M4439, Sigma), 1∶500 rabbit anti-LONP1 (#NBP1-81734, Novus Biologicals), 1∶500 rabbit anti-TFAM (#ab47548, Abcam), 1∶1000 rabbit anti-Hsp60 (#4870S, Cell Signaling Technology), 1∶1000 mouse anti-VDAC (#MSA03, MitoSciences), 1∶2000 mouse anti-OxPhos Complex V subunit β (#A21351, Molecular Probes/Life Technologies), 1∶1000 mouse anti-NDUFS3 (#ab14711, Abcam), 1∶3000 mouse anti-PDH (#MSP07, MitoSciences), 1∶50,000 mouse anti-Actin (#MAB1501, Chemicon/Bioscience Research Reagents). The rabbit anti-Mfn had been described previously [50]. The secondary antibody anti-mouse HRP (Sigma) was used at 1∶2500 for anti-Myc; 1∶10,000 for anti-VDAC and anti-NDUFS3; and 1∶7500 for anti–Complex V β, anti-PDH, and anti-Actin. The secondary antibody anti-rabbit HRP (Sigma) was used at 1∶10,000. Signal was detected using Thermo Scientific electrochemiluminescence reagents. Densitometry measurements of the western blot images were measured blind to genotype and condition using Fiji software [51]. Normalized western blot data were log-transformed when necessary to stabilize variance before means were compared using Student *t* test. Each experiment was performed at least three times.

### Flow cytometry analysis

Measurement of mitochondrial membrane potential in neural cells was conducted as previously described [28], except that the dissections were conducted in DME/Ham's F-12 High Glucose media without phenol red (Sigma) with 20 mM HEPES (Sigma), 2.5 mM glutamine (Sigma), and 0.5% trypsin (Invitrogen) at 25°C. Briefly, four adult *Drosophila* brains per genotype were dissected, dissociated at 25°C, and labeled with 10 nM TMRE at room temperature (Enzo Life Sciences). Samples were maintained at room temperature, in supplemented media with 10 nM TMRE, until flow cytometry analysis was performed. The effect of mitochondrial depolarization on TMRE accumulation was assessed by pretreating neural preps with 100 µM carbonyl cyanide m-chlorophenyl hydrazone (CCCP) for 10 minutes prior to the addition of TMRE. Flow cytometry was performed using a BD FACSCanto or a BD LSRII (BD Biosciences), equipped with a 635 nm laser. The mean TMRE fluorescence of each experimental sample was normalized to the mean fluorescence of the control sample prepared and analyzed on the same day.

### Mitochondrial fractionation

Heads from 30 male and 30 female flies were manually homogenized with a pestle in isolation buffer (220 mM mannitol, 68 mM sucrose, 20 mM HEPES pH 7.4, 80 mM KCl, 0.5 mM EGTA, 2 mM Mg(CH_3_COO)_2_) containing a protease inhibitor cocktail (#P8340, Sigma). The homogenate was centrifuged at 1500 *g* for 5 minutes at 4°C to prepare a postnuclear supernatant. The postnuclear supernatant was then subjected to a further round of centrifugation at 10,000 *g* for 25 minutes at 4°C to pellet mitochondria. The mitochondrial pellet was then either suspended in isolation buffer without protease inhibitors and used in protease protection experiments (see below), or solubilized in SDS-PAGE sample buffer and used in western blot analysis along with the supernatant fractions.

### Protease protection assay

Mitochondrial fractions in isolation buffer without protease inhibitors (prepared as described above) were divided in half. One half received a specific concentration of Proteinase K (ProK) (#19131, Qiagen) and the other half received an equal volume of buffer lacking ProK. These samples were incubated on ice for 20 minutes, followed by the addition of phenylmethylsulfonyl fluoride (PMSF) to inhibit ProK. SDS-PAGE sample buffer was then added to the samples, which were then boiled and used in western blot analysis. For experiments involving Triton X-100, equal amounts of ProK were added to both halves of the mitochondrial fraction. Following ProK addition, 1% Triton X-100 was added to one of the two samples, and both samples were incubated for 40 minutes on ice, followed by the addition of PMSF to inhibit ProK. SDS-PAGE sample buffer was then added to the samples, and the samples were boiled and used in western blot analysis.

### Immunohistochemistry

Adult thoracic muscle was dissected and then fixed in 4% paraformaldehyde in PBS. The tissue was blocked for one hour in PBS with 0.1% Triton X-100 and 10% fetal bovine serum, then incubated overnight in 1∶500 rabbit anti-GFP (#A11122, Life Technologies) and either 1∶500 mouse anti-Myc 9E10, 1∶500 mouse anti-FLAG (#F3165, Sigma), or 1∶2000 mouse anti-Cytochrome C (Cyto C) (#556433, BD Biosciences). The tissue was then washed in PBS with 0.1% Triton X-100 and incubated overnight with 1∶500 anti-rabbit Alexa 488 secondary antiserum (#A11034, Life Technologies) and 1∶500 (1∶1000 for Cyto C) anti-mouse Alexa 568 secondary antiserum (#A11031, Life Technologies). After final washes, the tissue was mounted in Prolong Gold (#P36934, Invitrogen) and imaged sequentially with 488 nm and 561 nm lasers on an Olympus FluoView FV1200 (Olympus America) with a 60× oil objective and 15× digital zoom, taken at 1024×1024 pixels and 30 steps of 0.13 µm.

### Image analysis

Each stack of 30 images was deconvoluted using Huygens Professional 4.4.0-p8 software (Scientific Volume Imaging), using a signal to noise ratio of 20 for the green channel and 18 for the red channel. Co-localization was calculated using the advanced Object Analyzer of the Huygens software, using a threshold of 270 or 2 times the standard deviation of the image, whichever was higher. The seed threshold for objects was set at 2% and the garbage threshold for objects at 500 voxels. At least 6 image stacks were analyzed per condition.

### Eye severity score

Flies were assigned to one of three phenotypic categories by an investigator blinded to genotype. Each fly was scored according to the severity of the more greatly affected eye. The three categories were as follows: mild, which ranged from completely WT appearance to bristle misorientation and slight deviation in ommatidial row arrangements; moderate, which consisted of overall ommatidial disorganization and mildly to moderately decreased eye size; and severe, which ranged from greatly reduced eye size to completely absent eyes. The mild, moderate and severe categories were assigned scores of one, two, and three, respectively, and the mean score for each genotype was calculated. Means were compared using Student *t* test.

### Thoracic indentation frequency

Flies were individually examined under a dissecting microscope for the presence of thoracic indentations as previously described [32]. Flies with indentations were assigned a score of one. Flies with no indentations were assigned a score of zero. The mean scores of the experimental and control (sibling) genotypes were compared by Student *t* test.

## Supporting Information

Figure S1 PINK1 mRNA levels in flies bearing one copy of the *PINK1-myc* transgene. qPCR of reverse transcribed RNA from control animals and those carrying one copy of the genomic *PINK1-myc* transgene was performed using two different sets of *PINK1* primers and compared to the internal housekeeping control transcript of *Rap2l*. PCR was performed at least three times. Error bars represent s.e.m.
(TIF)

Figure S2 Four PINK1 isoforms are detected in control flies, the smallest of which is less abundant in AFG3L2-deficient flies. (A) A long exposure of a western blot performed on head protein samples from flies bearing a Myc-tagged PINK1 genomic construct and either a control RNAi targeting *mCherry* (Control-R) or *AFG3L2*-RNAi1 (AFG3L2-R1) driven by *elav-GAL4*. Actin was used as a loading control. Four bands were detected in the control sample using an anti-Myc antibody and were named according to the protease that appears responsible for their production: FL-PINK1 for unprocessed PINK1; MPP-PINK1 for PINK1 processed by MPP; Rho-PINK1 for PINK1 processed by Rho-7/PARL; and AFG-PINK1 for PINK1 processed by AFG3L2. (B) Densitometry of the AFG-PINK1 band was performed using Fiji software and the band intensity was normalized to the Actin loading control. This ratio was then normalized to the Control-R AFG-PINK1/Actin ratio. AFG-PINK1 abundance is significantly reduced in AFG3L2-R1 flies. Experiments were repeated at least three times. Error bars represent s.e.m. *p<0.05 by Student *t* test.
(TIF)

Figure S3 Mitochondrial DNA levels are not altered by Lon deficiency. qPCR to measure mitochondrial and nuclear DNA levels was performed on DNA extracted from the heads of flies expressing PINK1-Myc and either Control-R, Lon-R1, or Lon-R2 driven by *elav-GAL4*. The qPCR was performed at least three times. Error bars represent s.e.m.
(TIF)

Figure S4 Disruption of mitochondrial membranes renders imported mitochondrial proteins sensitive to Proteinase K. (A) Mitochondrial fractions from the heads of flies expressing Control-R or Lon-R2 were prepared and divided in half. Each sample was treated with 0.5 µg/ml Proteinase K (ProK), and 1% Triton X-100 was added to one of the two samples from each genotype, as indicated. After incubation, samples were subjected to western blot analysis using antibodies to the indicated proteins. (B) Quantification of the PINK1-Myc, Comp V β, and PDH bands from experiments represented by panel A was performed by densitometry using Fiji software. Ratios of the band intensities in samples treated with Triton X-100 relative to samples without Triton X-100 are shown for the indicated proteins. Experiments were repeated at least three times. Error bars represent s.e.m.
(TIF)

Figure S5 Eye phenotypes of flies expressing RNAi constructs targeting *Lon* in a WT background. Neither (A) the control RNAi (Control-R) nor the *Lon* RNAi constructs, (B) Lon-R1 or (C) Lon-R2, resulted in a rough eye phenotype when expressed using the *ey-GAL4* driver in an otherwise wild-type background.
(TIF)

Figure S6 Mutation in *Lon* does not suppress a *PINK1* null phenotype. No significant suppression of the thoracic indentation frequency of *PINK1^B9^* null mutants was observed in *Lon* heterozygotes: *PINK1^B9^* (*n* = 23), *PINK1^B9^*; *Lon^EP^*/+ (*n* = 25). Error bars represent s.e.m.
(TIF)

Table S1 List of protease genes and RNAi constructs used in screen.
(DOCX)
