# Supplementary figures and images for: PINK1-Parkin Pathway Activity Is Regulated by Degradation of PINK1 in the Mitochondrial Matrix
Source: PLoS Genet. 2014 May 29;10(5):e1004279. doi: 10.1371/journal.pgen.1004279 (PMC4038460; doi:10.1371/journal.pgen.1004279)

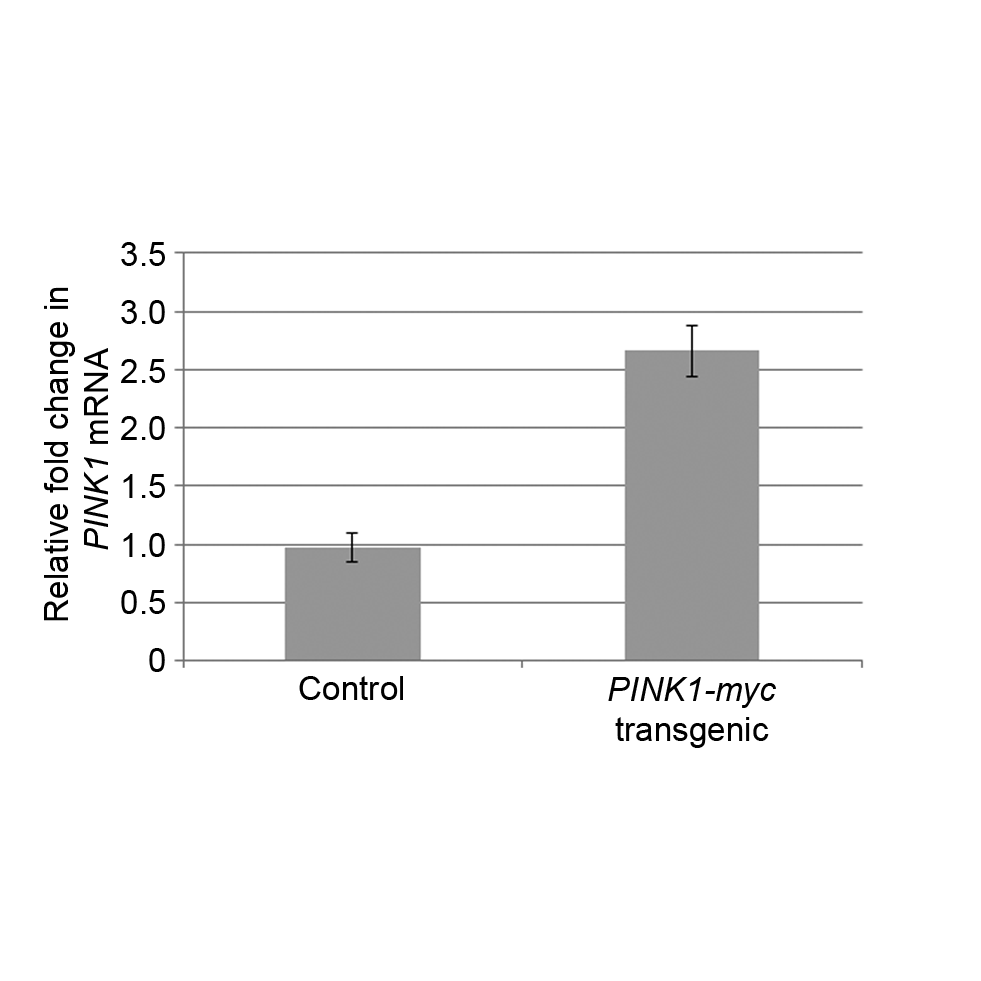

Supplement: Figure S1 — PINK1 mRNA levels in flies bearing one copy of the PINK1-myc transgene. qPCR of reverse transcribed RNA from control animals and those carrying one copy of the genomic PINK1-myc transgene was performed using two different sets of PINK1 primers and compared to the internal housekeeping control transcript of Rap2l. PCR was performed at least three times. Error bars represent s.e.m. (TIF) [file pgen.1004279.s001.tif]

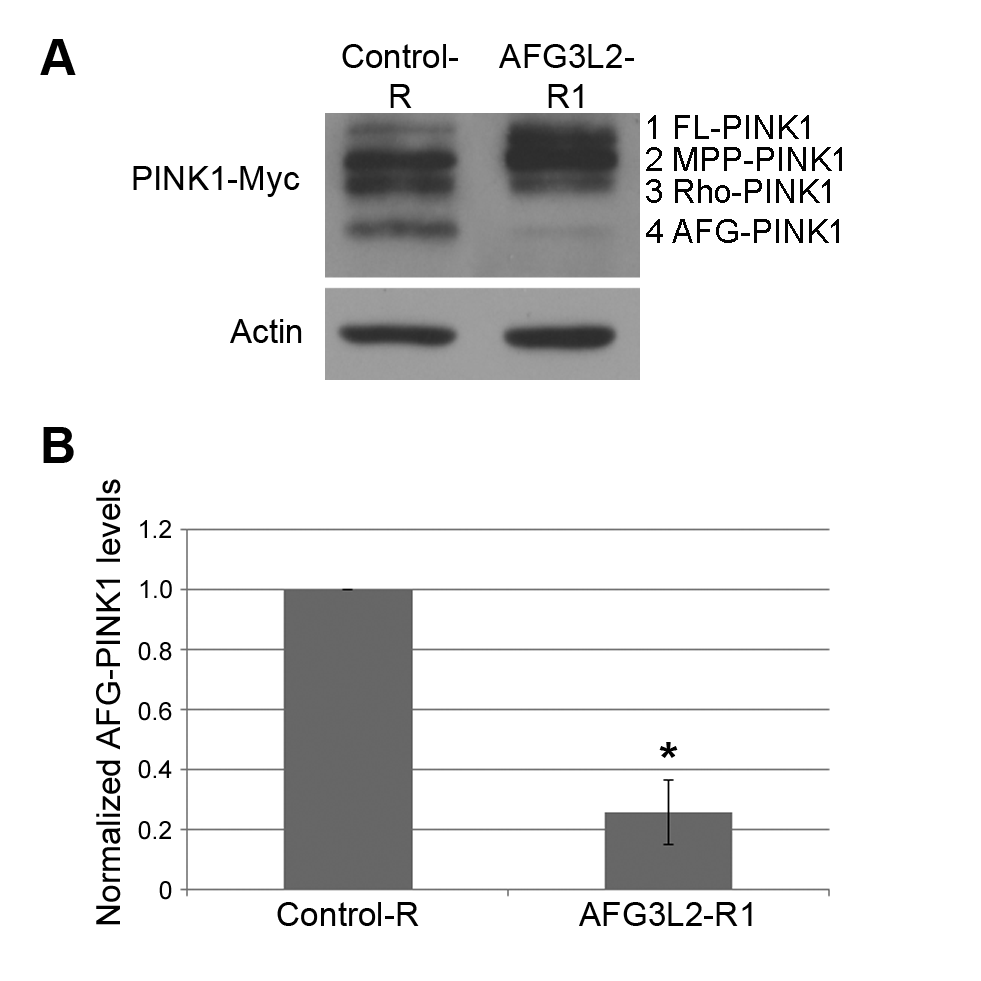

Supplement: Figure S2 — Four PINK1 isoforms are detected in control flies, the smallest of which is less abundant in AFG3L2-deficient flies. (A) A long exposure of a western blot performed on head protein samples from flies bearing a Myc-tagged PINK1 genomic construct and either a control RNAi targeting mCherry (Control-R) or AFG3L2-RNAi1 (AFG3L2-R1) driven by elav-GAL4. Actin was used as a loading control. Four bands were detected in the control sample using an anti-Myc antibody and were named according to the protease that appears responsible for their production: FL-PINK1 for unprocessed PINK1; MPP-PINK1 for PINK1 processed by MPP; Rho-PINK1 for PINK1 processed by Rho-7/PARL; and AFG-PINK1 for PINK1 processed by AFG3L2. (B) Densitometry of the AFG-PINK1 band was performed using Fiji software and the band intensity was normalized to the Actin loading control. This ratio was then normalized to the Control-R AFG-PINK1/Actin ratio. AFG-PINK1 abundance is significantly reduced in AFG3L2-R1 flies. Experiments were repeated at least three times. Error bars represent s.e.m. *p<0.05 by Student t test. (TIF) [file pgen.1004279.s002.tif]

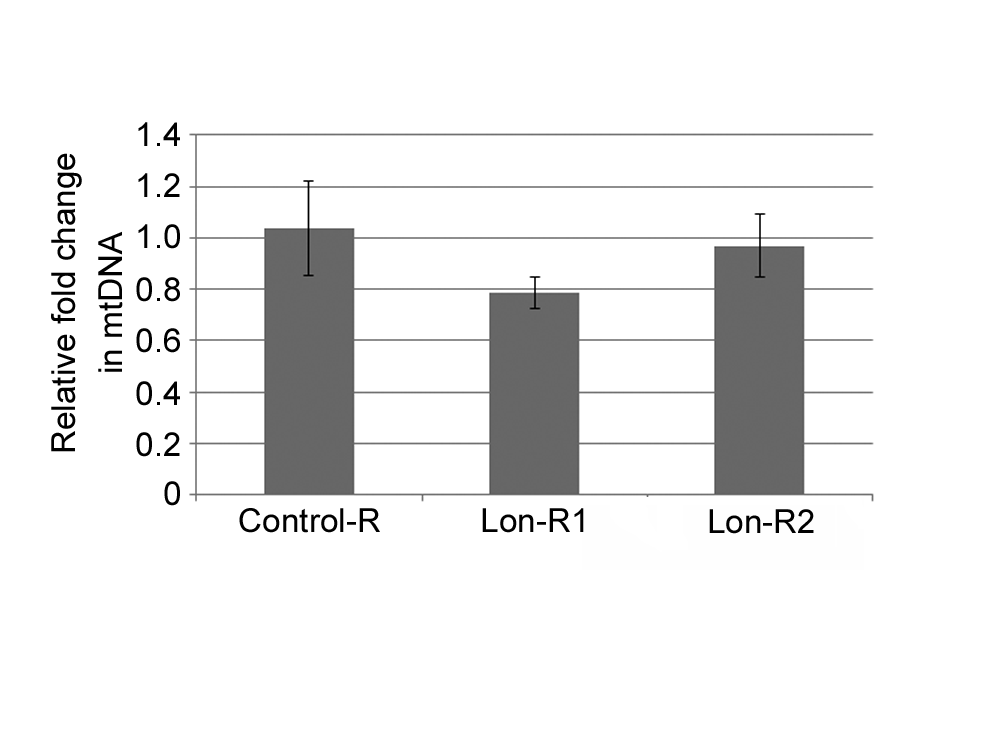

Supplement: Figure S3 — Mitochondrial DNA levels are not altered by Lon deficiency. qPCR to measure mitochondrial and nuclear DNA levels was performed on DNA extracted from the heads of flies expressing PINK1-Myc and either Control-R, Lon-R1, or Lon-R2 driven by elav-GAL4. The qPCR was performed at least three times. Error bars represent s.e.m. (TIF) [file pgen.1004279.s003.tif]

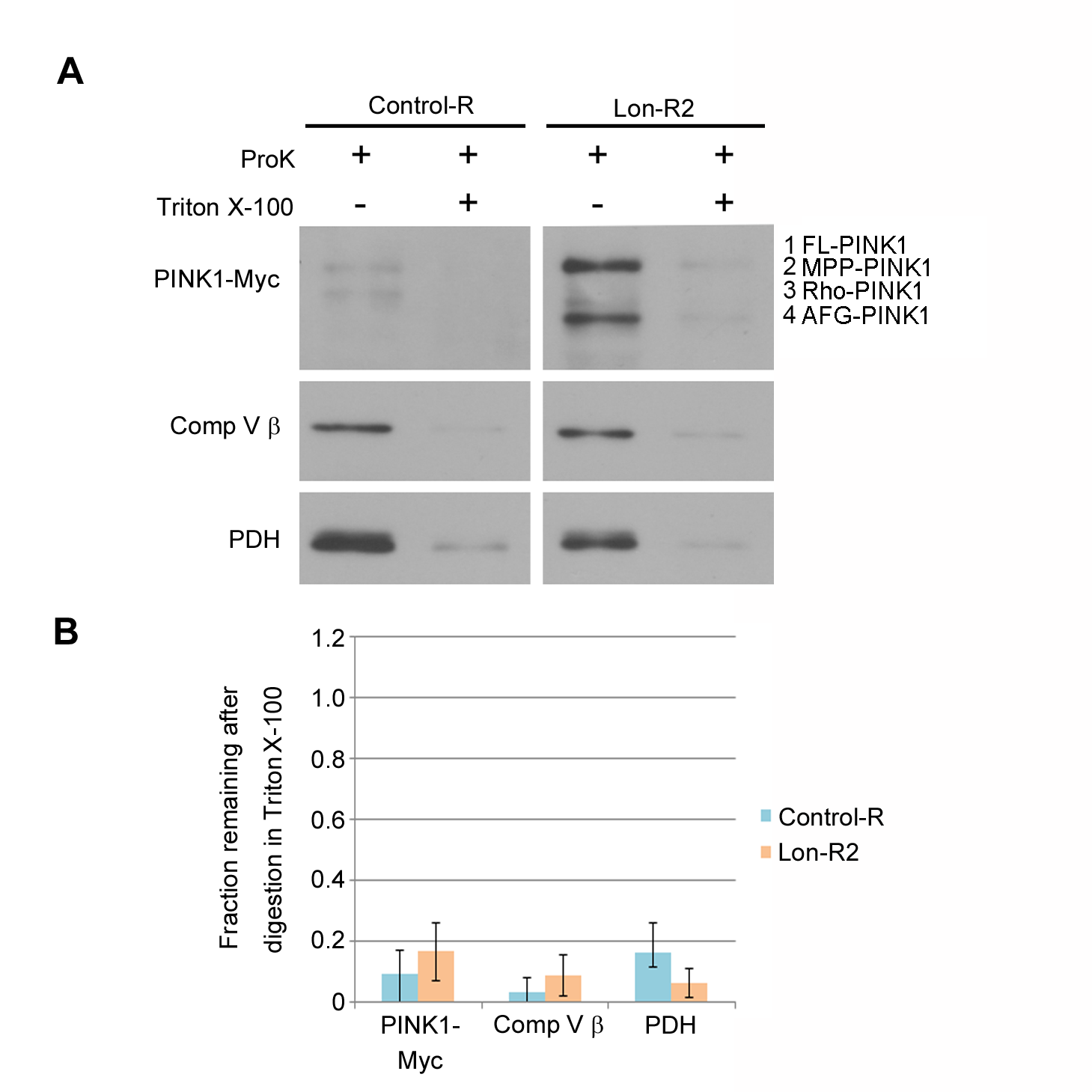

Supplement: Figure S4 — Disruption of mitochondrial membranes renders imported mitochondrial proteins sensitive to Proteinase K. (A) Mitochondrial fractions from the heads of flies expressing Control-R or Lon-R2 were prepared and divided in half. Each sample was treated with 0.5 µg/ml Proteinase K (ProK), and 1% Triton X-100 was added to one of the two samples from each genotype, as indicated. After incubation, samples were subjected to western blot analysis using antibodies to the indicated proteins. (B) Quantification of the PINK1-Myc, Comp V β, and PDH bands from experiments represented by panel A was performed by densitometry using Fiji software. Ratios of the band intensities in samples treated with Triton X-100 relative to samples without Triton X-100 are shown for the indicated proteins. Experiments were repeated at least three times. Error bars represent s.e.m. (TIF) [file pgen.1004279.s004.tif]

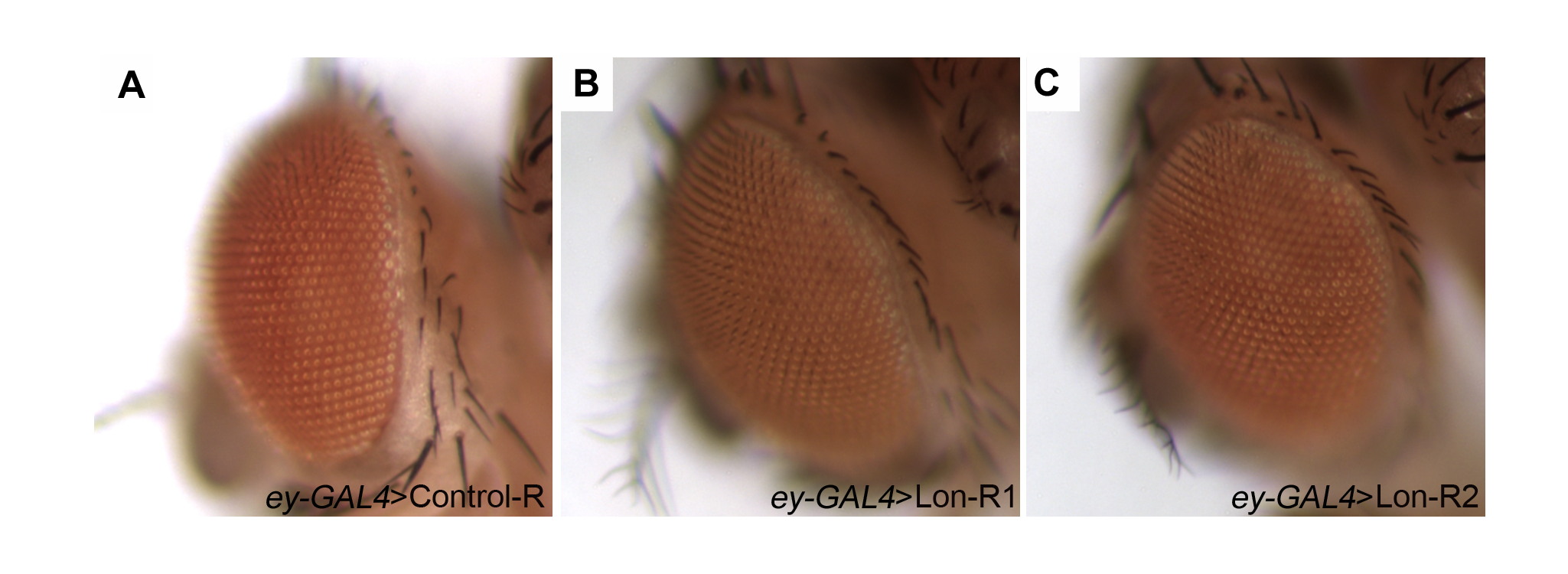

Supplement: Figure S5 — Eye phenotypes of flies expressing RNAi constructs targeting Lon in a WT background. Neither (A) the control RNAi (Control-R) nor the Lon RNAi constructs, (B) Lon-R1 or (C) Lon-R2, resulted in a rough eye phenotype when expressed using the ey-GAL4 driver in an otherwise wild-type background. (TIF) [file pgen.1004279.s005.tif]

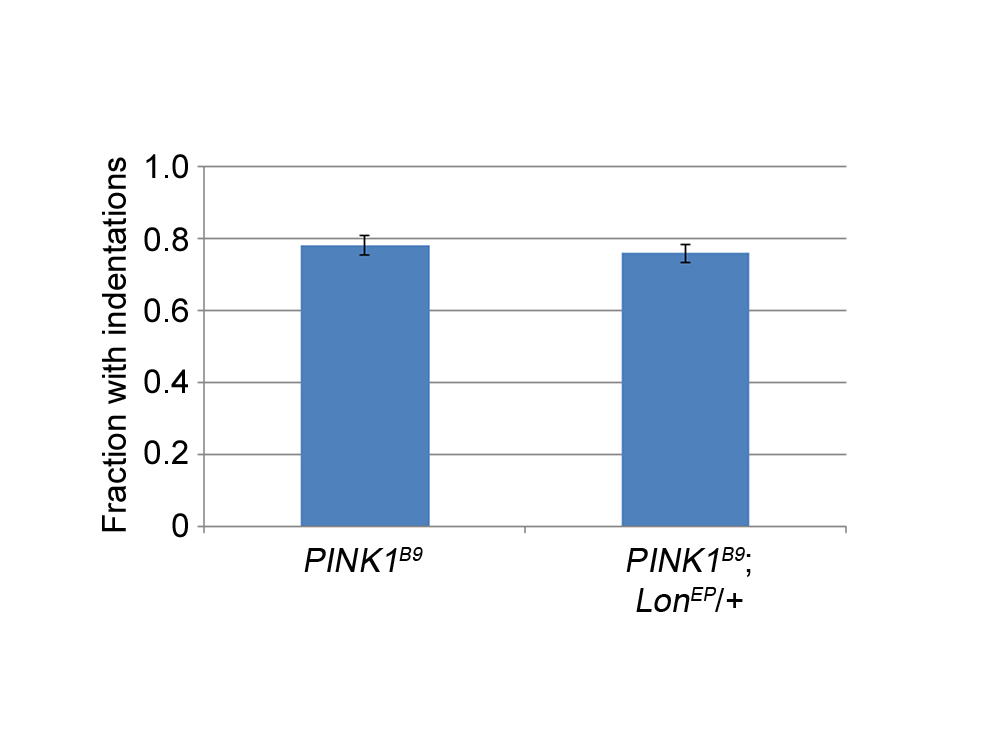

Supplement: Figure S6 — Mutation in Lon does not suppress a PINK1 null phenotype. No significant suppression of the thoracic indentation frequency of PINK1B9 null mutants was observed in Lon heterozygotes: PINK1B9 (n = 23), PINK1B9; LonEP/+ (n = 25). Error bars represent s.e.m. (TIF) [file pgen.1004279.s006.tif]
