# Supplementary material for: PINK1-Parkin Pathway Activity Is Regulated by Degradation of PINK1 in the Mitochondrial Matrix
Source: PLoS Genet. 2014 May 29;10(5):e1004279. doi: 10.1371/journal.pgen.1004279 (PMC4038460; doi:10.1371/journal.pgen.1004279)
Supplement: Table S1 — List of protease genes and RNAi constructs used in screen. (DOCX) [file pgen.1004279.s007.docx]

Table S1. List of protease genes and RNAi constructs used in screen.

| **Drosophila gene** | **Human homolog** | **RNAi reagent(s)** | **Effect on PINK1** |
| --- | --- | --- | --- |
| CG6512 | AFG3L2 | V: 109629 & 8515 | Accumulation |
| CG4908 | BCSIL | BL: 31074^D^ & 31075^D^ | None |
| CG5045 | CLPP | V: 103423 & 26688 | None |
| CG4538 | CLPX | V: 39699 | None |
| CG9240 | IMMP1L | V: 103596 | None |
| CG8798 | LONP1 | BL: 34586; V: 36036 | Accumulation |
| CG7791 | MIPEP | V: 109658 | None |
| rhomboid-7 | PARL | BL: 35617; V: 108343 & 45847 | None |
| CG3107 | PITRM1 | V:103826 | None |
| CG8728 | PMPCA | BL: 34074 | ND (lethal) |
| CG2658 | SPG7 | BL: 31100^D^ & 31223^D^ | None |
| CG9581 | XPNPEP3 | BL: 34547 | None |
| CG3499 | YME1L1 | V: 105143 & 34282 | None |

^D^with Dicer present

BL = Bloomington Drosophila Stock Center

V = Vienna Drosophila Resource Center
